# Supplementary material for: Improved ACOM pattern matching in 4D-STEM through adaptive sub-pixel peak detection and image reconstruction
Source: Sci Rep. 2024 May 29;14:12385. doi: 10.1038/s41598-024-63060-5 (PMC11137144; doi:10.1038/s41598-024-63060-5)
Supplement: Supplementary file 1 — Supplementary Information. [file 41598_2024_63060_MOESM1_ESM.docx]

**Supporting information:**

**Adaptive Diffraction Image Registration**

**for 4D-STEM to optimize ACOM Pattern Matching**

Nicolas Folastre^1,4^, Junhao Cao^1,4^, Gozde Oney^1,2,4^, Sunkyu Park^1,2^,
Arash Jamali^1^, Christian Masquelier^1,4,5^, Laurence Croguennec^2,4,5^,
Muriel Veron^3^, Edgar F. Rauch^3^, Arnaud Demortière^1,4,5^*

^1^ *Laboratoire de Réactivité et Chimie des Solides (LRCS), CNRS-UPJV UMR 7314, Hub de l’Energie, rue Baudelocque, 80039 Amiens Cedex, France.*

^2^ *Institut de Chimie de la Matière Condensée de Bordeaux (ICMCB), Bordeaux*

^3^ *Univ. Grenoble Alpes, CNRS, Grenoble INP, SIMAP, F-38000 Grenoble*

^4^ *Réseau sur le Stockage Electrochimique de l’Energie (RS2E), CNRS FR 3459, Hub de l’Energie, rue Baudelocque, 80039 Amiens Cedex, France.*

^5^ *ALISTORE-European Research Institute, CNRS FR 3104, Hub de l’Energie, rue Baudelocque, 80039 Amiens Cedex, France*

Corresponding Author: [*arnaud.demortiere@cnrs.fr](mailto:*arnaud.demortiere@cnrs.fr)


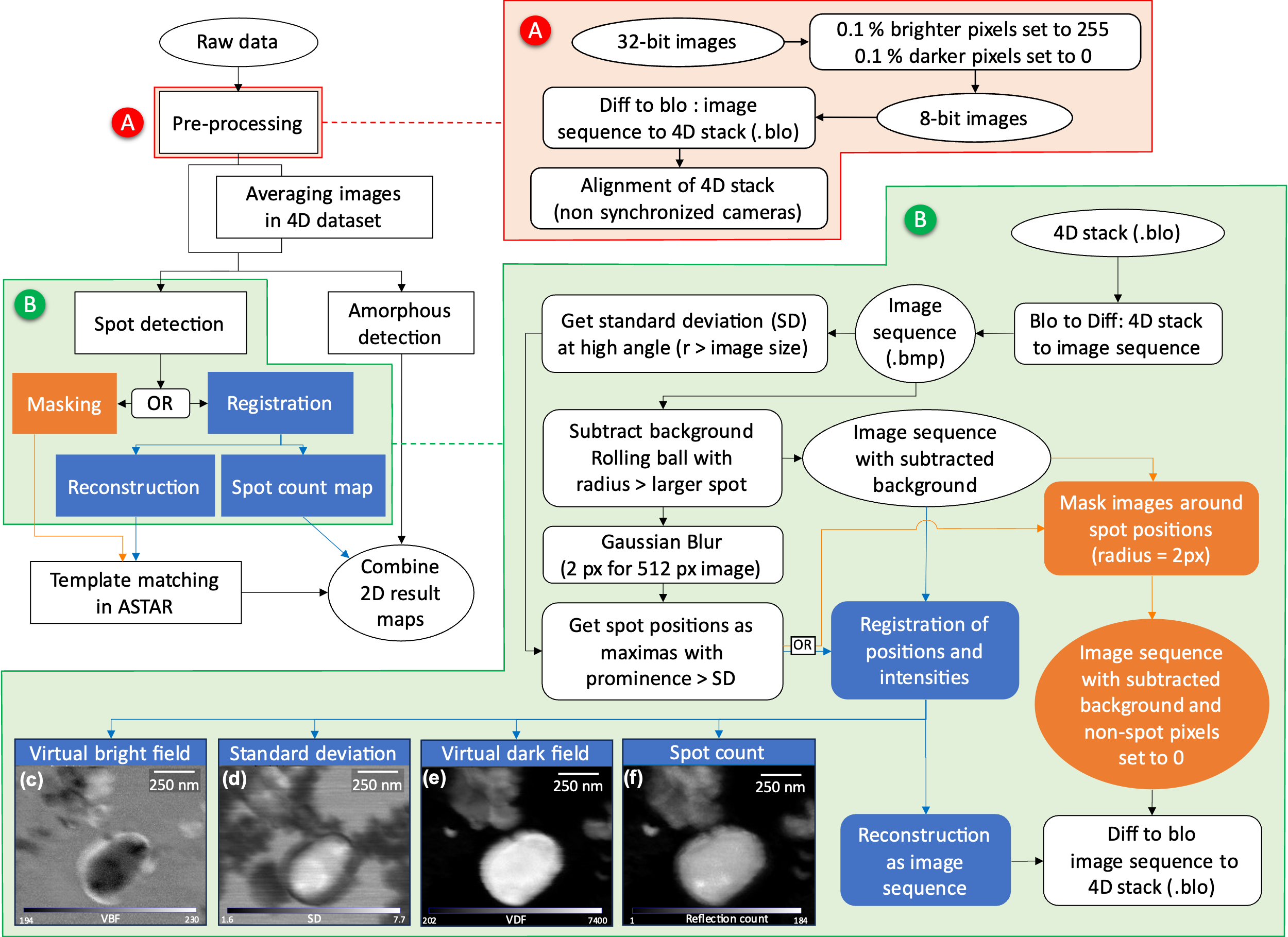


**Figure S 1** **Data preparation strategy applied to 4D-STEM data.** A) Pre-processing of experimental 4D-STEM data. B) The registration and reconstruction strategy is used to keep essential diffraction pattern information as reflection positions and intensities. The masking branch consists of directly using the spot positions to mask each diffraction pattern. C) A virtual brightfield is built integrating the intensity of the direct/central spot. D) The standard deviation map displays the standard deviation measured in the pattern at high angles. E) A virtual dark field is built integrating the intensity of all registered reflections without the direct/central spot. F) A spot count map is built by counting the number of reflections registered in each pattern.

**Filters**

To enhance the quality of diffraction images, the initial step involves processing operations among the scanned images. The primary technique employed is the "Mean" function (µ), which normalizes the combined values of nn adjacent images using a 3x3 kernel that slides across the scan. This process is governed by the following equation:

1

$$\mu=\frac{1}{n}\sum_{1}^{n} x_{i} n=9 (1)$$

The filter processes by averaging sets of nine neighboring images in a scan, producing a resultant scan of identical size in which each differential phase (DP) image is the average of these nine images. Utilizing an excessively large kernel, however, can significantly diminish the spatial resolution, contingent upon the scan's original spatial resolution. Consequently, opting for a larger scan size while maintaining a lower spatial resolution during acquisition can be an effective strategy to achieve comparable diffraction image quality.


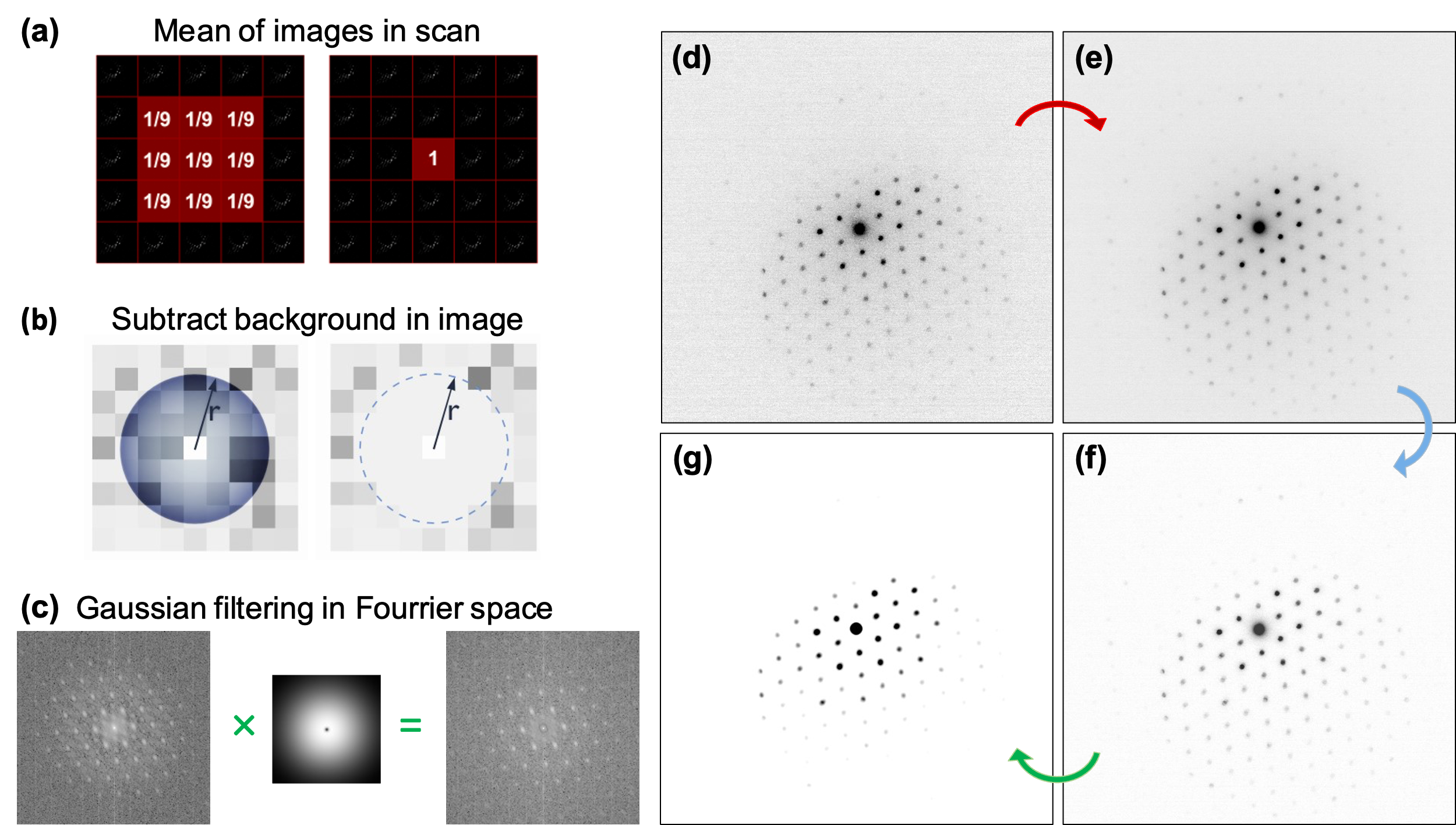


**Figure S 2 Intiale Filtering Strategy.** (a) Mean of DP images neighbors in the scan (b) Subtraction of the local background in DP images over a disc of radius r (c) Bandpass filters using Gaussian filtering in Fourier space. Diffraction Pattern (d) before filtering (e) after summing 9 DPs (f) with background subtracted and (g) with bandpass filter applied to keep a defined range of feature size.

To improve pattern matching in ACOM, we first aim to remove the noise contained between spots of the diffraction pattern. To complete this specific task, imaging tools are used that allow removing the background due to scattering effects from the image or highlighting objects of a certain size in the image as reflections in a diffraction pattern. Consequently, the second filter is the "Subtract Background Rolling Ball". The method is shown in **Figure S3**.


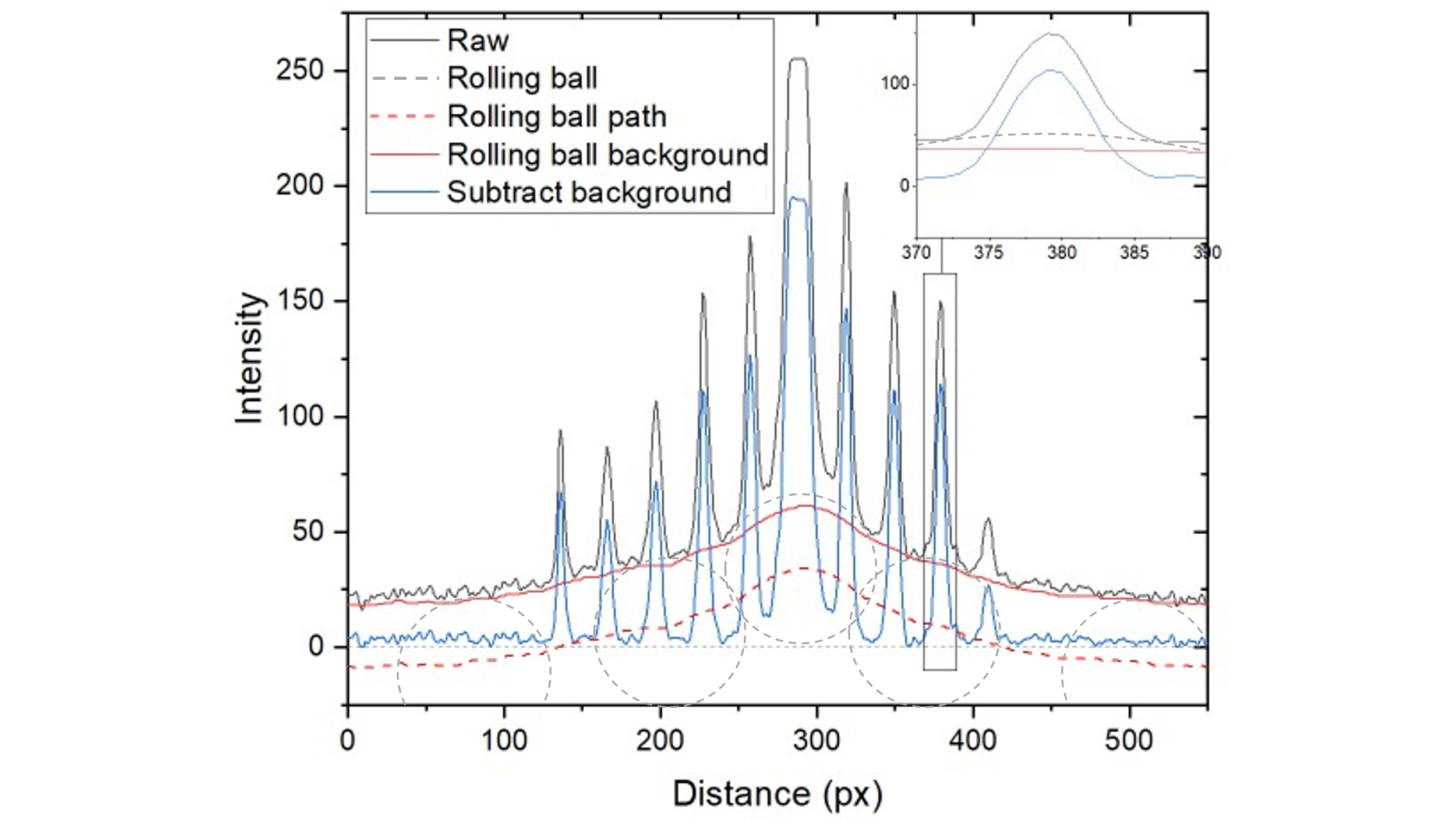


**Figure S 3** **Subtract Background “Rolling ball“ method.** The rolling ball slides under the signal curve and trace a path. This path is shifted by the radius of the ball to trace the background. Finally the background is subtracted from the signal. Note that this radius of rolling ball is too large, it should be smaller to be slightly bigger than the central spot size.

When the difference between the raw signal and the background is negative, the pixel value is set to zero. This method significantly reduces variations across large areas, resulting in a more uniform image background. The technique effectively minimizes the scattering effect in diffraction images while preserving the diffraction signal. To achieve this, it is essential to use a filter with a kernel size (r) that is larger than the size of the objects in the signal. This prevents the background values from being overly affected by high intensity values and broad peak widths. By adhering to this approach, the background estimation remains precise even near large peaks and is only moderately influenced by high peak values, such as the central peak, as illustrated in the background profile shown in **Figure 4**. This filter has been widely used in powder diffraction and in 2D on high-resolution - electron backscatter diffraction (HR-EBSD) images^45^, in particular to characterize its angular dependence.^46^

The “Bandpass” filter aims to highlight features in the image whose size is included in a defined range. A fast Fourier transform (FFT) is applied to the image, which is multiplied by a Gaussian filter (**Equations 2, 3**). The reversed FFT of the result gives the filtered image.

2

$${\left( FFT \right) f}_{j}=\sum_{k=0}^{n-1} x_{k}e^{\frac{-2\pi i}{n}jk} j=0,\ldots,n-1 (3)$$

3

$g\left( x \right)= \frac{1}{\sqrt{2\cdot\pi}\cdot\sigma}{\cdot e}^{-\frac{x^{2}}{2\sigma^{2}}} (4)$

This method enhances features whose size is covered by the bandpass filter. We typically take the size of the central spot as the upper limit and the lower size is limited to 2 pixels (px) to filter the remaining noise. The bandpass filter acts as both a high pass filter and a low pass filter, thus it respectively reduces the noise consisting of high spatial frequencies but also limits the "spreading" of objects in the image.

Indeed, the low pass filter decreases noise but attenuates the details of the image, which manifests itself as a more pronounced blur in the filtered image. On the other hand, the high pass filter emphasizes contours and image detail but amplifies noise.^47^

In practice, the bandpass filter consists of a high pass filter followed by a low pass filter. The large structures of the image are filtered with a high-pass filter, the limit of which must correspond to the maximum size of the object to be preserved, which corresponds in the diffraction images to the size of the central spot. Small image structures such as indistinguishable noise from a tiny spot are filtered out by a low pass filter which should be set to the size of the smallest discernible object in the noise. Thus, if the two parameters of the band-pass filter are well adjusted, it is possible to keep the object sizes corresponding to the spots contained in the image, by limiting the addition of blurring or noise.

In addition, a threshold has to be applied to the resulting image to conserve the 1% of brighter pixels corresponding to the reflections. However, it is important to note that the FFTs on the relatively noisy diffraction images induce artifacts that are difficult to eliminate by a simple threshold, as shown in **Figure S2**. Consequently, the use of such a filter is only recommended on data with very little noise and/or with sufficient contrast.

The order in which filters are applied always remains the same. We apply the "Mean" filter to gain signal-to-noise (SNR) by averaging very close original images (< 20 nm between two DPs). The "Subtract Background" filter must be applied before any registration or filter to demarcate the spots from the large background. Finally, the "Bandpass" filter is applied last to enhance the diffraction spots depending on their size. These filters were explored in this work initially to directly improve pattern-matching by filtering only, then were studied as a potential springboard or first step to facilitate the registration of reflections.

***Registration and reconstruction of a reflection***

***
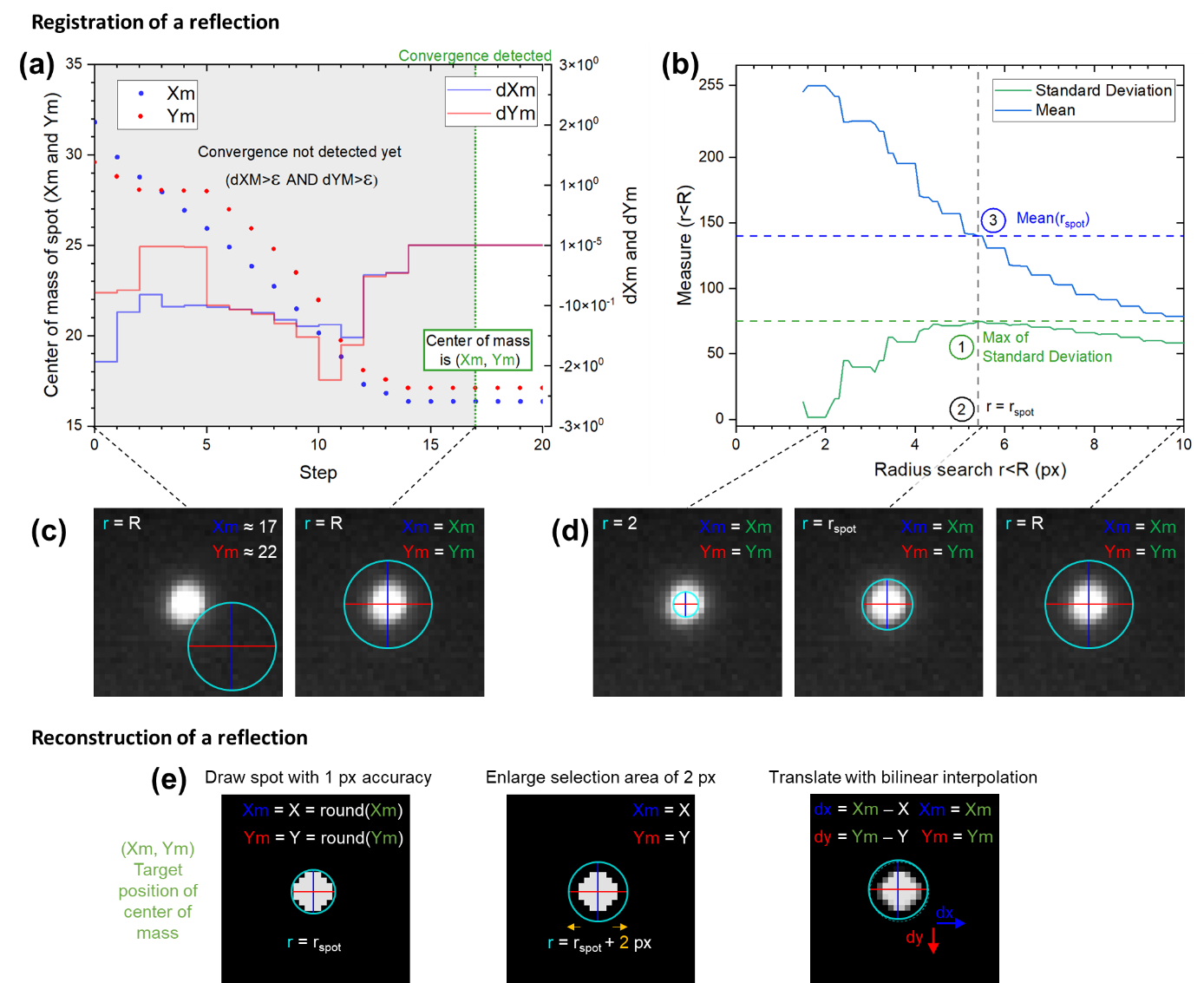
***

**Figure S 4** Feature extraction and reconstruction of a reflection. (a) Convergence of the reflection position (X, Y) toward the center of mass (Xm, Ym) of the reflection keeping a constant radius r=R, as illustrated on (c). Convergence is reached when the Center of mass is constant (Xm, Ym < 10-5). (b, d) Refinement of the radius of the reflection, starting from a minimum radius of 2 px, ending at a fixed value corresponding to the minimal distance between two spots in the dataset. The final radius corresponds to the highest measured SD in the sery minus 1 step (1 px). The intensity is taken as the mean measured at this radius. (e) Reconstruction is done in two steps : first the spot is drawn at a rounded position (at 1 px accuracy) of the exact one with the registered radius and intensity. Then, the selection is enlarged to operate a translation of the values using a bilinear interpolation, thus displacing the center of mass to the exact registered position in the reconstructed image.

***Noise Study***


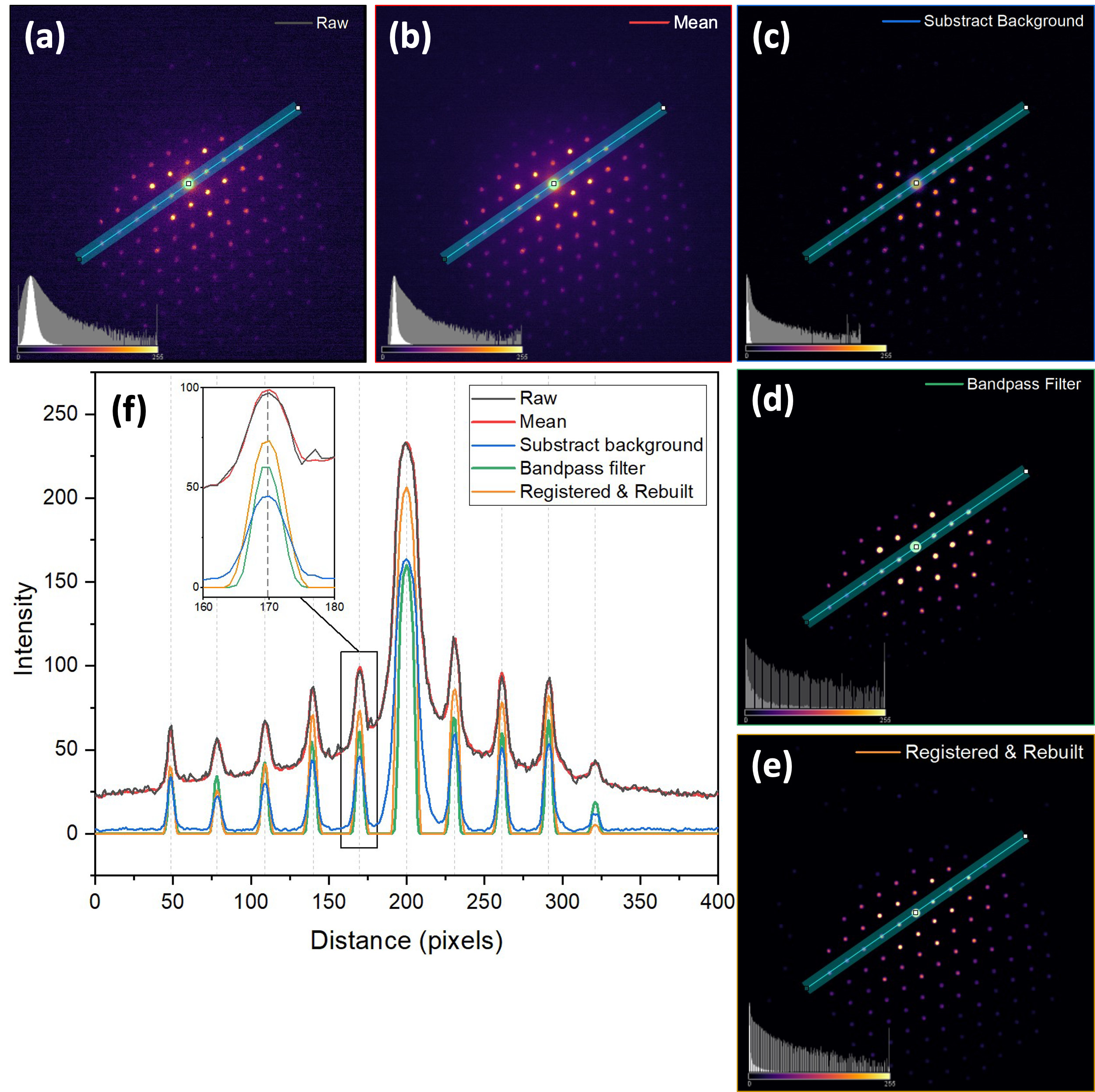


**Figure S 5** Profile of a DP with (a) no filter (b) mean filter over 9 DPs (c) subtracted backgroud with a radius of 25 px (d) Bandpass filter with range of 2 to 25 px. (e) Reconstruction using registration of subpixel-accurate positions, radius, and intensity of reflections (f)Plot of corresponding intensity profiles.

By applying these filters, we try to remove the noise and the background contribution due to scattering effects, with a limited alteration of the relative intensity between the spots. The objective is to eventually avoid noise overfitting with the simulated DPs during the cross-correlation calculation. By plotting the profile of the same DP at different filtering levels as shown in **Figure S5**, we observe a clear decrease in the height under the curve by using the "Subtract background". Then the "Bandpass" filter reduces the remaining background by keeping only the spots filtered by size. In parallel, at each level of filtering, the histogram of the image evolves, and we observe larger populations of low gray levels by filtering with the "Mean", which initially stretches the histogram while preserving the signals close in intensity, then with the "rolling ball subtract background " which reduces the background towards the darkest levels of the current image. Finally, the "Bandpass" filter increases the values on the spot positions and brings the gray level of the other pixels to 0, which results in a gradient of gray levels in the immediate environment of the spots and very strong dark populations. When the bandpass filter is used, a final threshold is applied to cut off the lowest gray levels and consequently assign the value 0 to these pixels. Filtering by bandpass filter before registration was considered and then discarded because it induces consequent artifacts and requires the use of a threshold that is difficult to quantify, as shown in **Figure S6**.


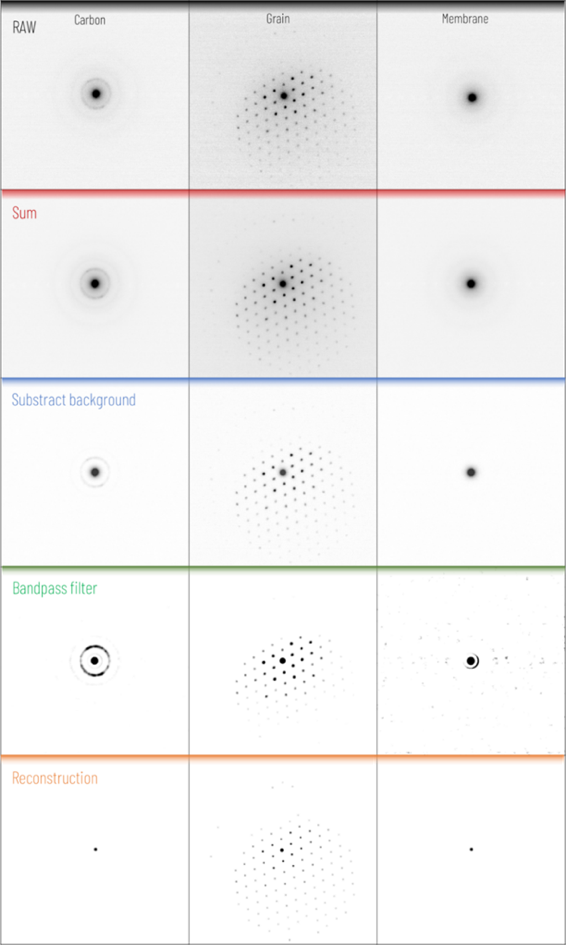
Since the errors are squared before they are averaged, the RMSE gives a relatively high weight to large errors. This means the RMSE should be more useful when large errors are particularly undesirable. MAE and SNR are not used in this work, as PSNR is more adapted than SNR to measure the variation of power of intensity changes, and MAE is less and less used because of its lack of normalization and representativity of general changes compared to other metrics.

**Figure S 6** Evolution of example images of amorphous carbon, crystalline grain and membrane only areas through filtering and reconstruction. Two main points can be observed: (1) the not yet released features for amorphous ring detection in the reconstruction process result in the absence of the amorphous rings in the reconstructed dataset, (2) The reconstructed dataset has a lower background (values are 0) and more signal-over-noise than any other filters presented here, positive values all being true positives.

$$\mathrm{SNR}=10\cdot{log}_{10}\left[ \frac{\sum_{0}^{n_{x}-1} \sum_{0}^{n_{y}-1} \left[ r\left( x, y \right) \right]^{2}}{\sum_{0}^{n_{x}-1} \sum_{0}^{n_{y}-1} \left[ r\left( x, y \right)-t\left( x, y \right) \right]^{2}} \right] (9)$$

$$\mathrm{PSNR}=10\cdot{log}_{10}\left[ \frac{{max\left( r\left( x, y \right) \right)}^{2}}{\frac{1}{n_{x}n_{y}}\cdot\sum_{0}^{n_{x}-1} \sum_{0}^{n_{y}-1} \left[ r\left( x, y \right)-t\left( x, y \right) \right]^{2}} \right] (10)$$

$$\mathrm{RMSE}= \sqrt{\frac{1}{n_{x}n_{y}}\cdot\sum_{0}^{n_{x}-1} \sum_{0}^{n_{y}-1} \left[ r\left( x, y \right)-t\left( x, y \right) \right]^{2}} (11)$$

$$\mathrm{MAE}=\frac{1}{n_{x}n_{y}}\cdot\sum_{0}^{n_{x}-1} \sum_{0}^{n_{y}-1} \left[ r\left( x, y \right)-t\left( x, y \right) \right] (12)$$

$\mathrm{SSIM}\left( x,y \right)=l\left( x,y \right)\cdot c\left( x,y \right)\cdot$s$\left( x,y \right)=\frac{\left( 2\mu_{x}\mu_{y}+ c_{1} \right)\left( 2\sigma_{x}\sigma_{y}+ c_{2} \right)\left( {cov}_{xy}+ c_{3} \right)}{\left( \mu_{x}^{2}+\mu_{y}^{2}+ c_{1} \right)\left( \sigma_{x}^{2}+\sigma_{y}^{2}+ c_{2} \right)\left( \sigma_{x}\sigma_{y}+ c_{3} \right)} (13)$

**Figure S 7** Equations of Image quality metrics: (9) signal over noise ratio (SNR), (10) peak signal over noise ratio (PSNR), (11) root mean square error (RMSE) , (12) mean average error (MAE) and (13) structural similarity index measure (SSIM).

***
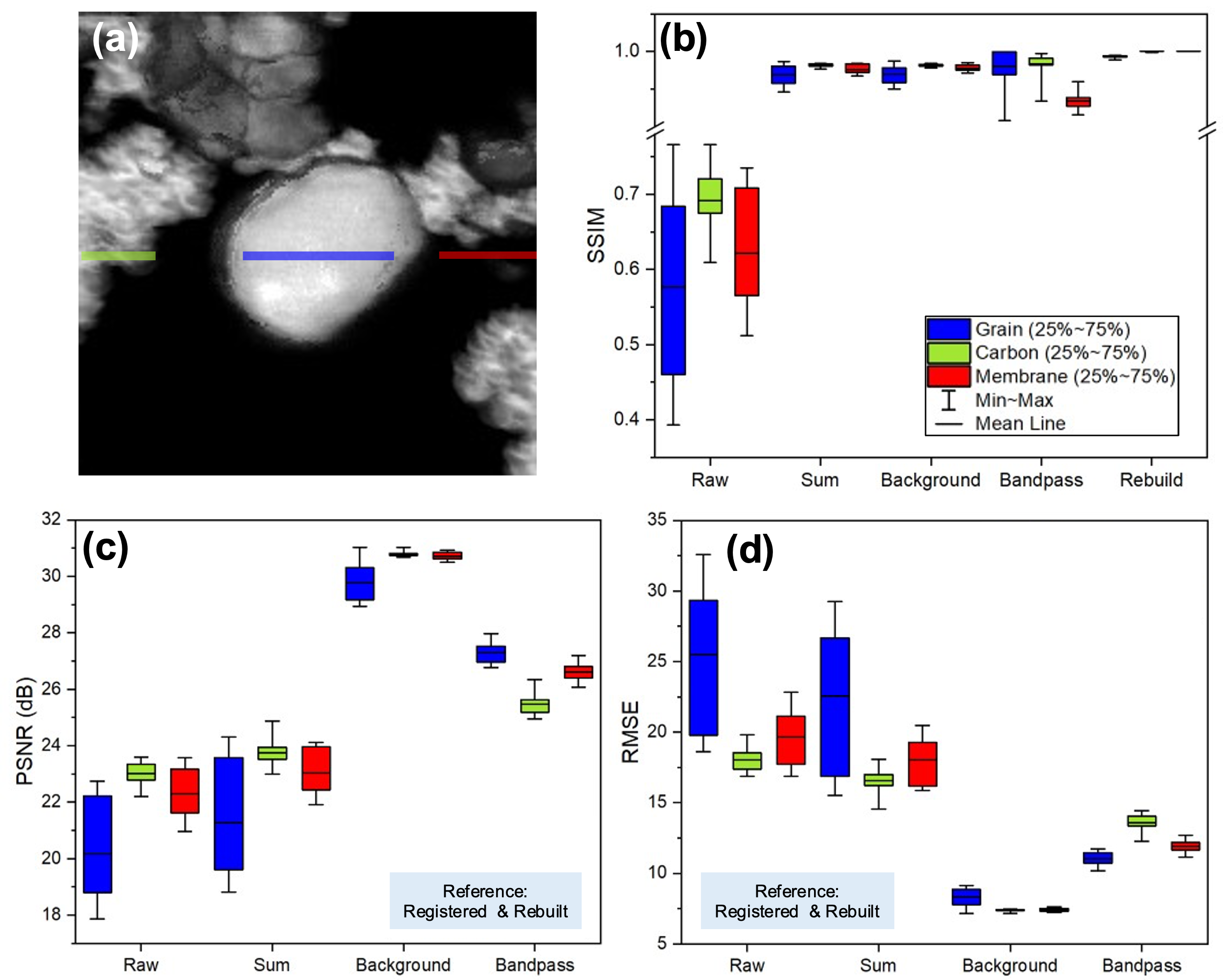
***

**Figure S 8** (a) Image quality metrics filtering different image types: amorphous carbon, crystalline grain, and substrate of the grid (i.e. thin carbon membrane). The metrics used are (b) Peak Signal-over-Noise (PSNR), (c) Structural similarity index measure (SSIM), and (d) Root-mean-square error (RMSE). The registered and reconstructed images as been set as references for all metrics but SSIM. More details on the choice of metrics can be found below **Figure S2**

The quality assessment of the images was measured according to different metrics allowing us to characterize the preservation of the structure of the image through the different filters, to detect the possible appearance of artefacts, or even to quantify the noise reduction. As shown in **Figure S8**, we have chosen to observe these behaviors on 3 types of content: the crystal diffraction images characterized by the presence of diffraction spots, the diffusion rings due to the presence of an amorphous phase, and finally the result of the passing beam only through the thin carbon membrane of the grid support.

The structural similarity index measure (SSIM) is applied by sliding stack on each dataset to indicate the structural similarity intrinsic to each method of filtering by the type of object observed. As shown in **Figure** **S8b**, we notice that the fact of averaging naturally increases this metric, which continues to improve slightly with the subtraction of the background which also subtracts noise. The reconstruction from the averaged and filtered data shows a significant increase in SSIM, as the majority of the image then contains -more than 95% of - black pixels and reflections relatively aligned in the axis of the stack.

However, the SSIM drops slightly when filtering by a bandpass filter. Indeed, this reduction is due to the formation of artifacts (**Figure S6**) which are more visible around the direct spot when it is very bright. More precisely, when we mainly observe the interaction of the beam with the membrane because only a few contributions are coming from the amorphous phase or a crystalline phase, there is more probability that the bandpass induces artifacts on the large saturated direct spot. The formation of these artifacts is technically due to the saturation of the histogram towards very high gray values which leaves very few gray levels for the threshold post-bandpass filter to be effective.

For the peak signal-over-noise-ratio PSNR and root-mean-square-error RMSE calculations, the reconstructed image was taken as the reference signal and not the original image. We show in **Figure S8c** the improvement in the pattern-matching results which justifies that the reconstructed diffraction signal serves as a reference here.. Thus, the quality of the signal is evaluated for each filtering step by focusing mainly on the reflections of the diffraction images. However, it is important to note that the reconstructed images optimize the diffraction signal in the form of spots, but not for the signal in the form of rings formed by the amorphous carbons present.

PSNR is normalized to signal dynamics and represents how close a processed image is to its original, and RMSE measures deviations, called errors, and grows noticeably and disproportionately with them.^48,49^ The RMSE is therefore a metric that makes it possible to better visualize the differences. As shown in **Figure S8c-d**, the operation of summing neighboring diffraction patterns significantly improves the diffracted signal-over-noise (SNR). Then subtraction of the background component has a more pronounced effect on the improvement of the diffraction pattern, thanks to the effect of the almost complete separation of the diffraction and diffusion components on the intensities of the reflections.

Note that this processing also radically reduces the standard deviation of the errors, which means that the diffraction images are more normalized among themselves as well, as can also be seen through the SSIM. Finally, the bandpass filter deteriorates the diffraction signal, because of the effects of saturation of the image which generates artifacts and also modifies the intensities of the diffraction peaks in an irreversible manner.

After applying the sum of the shots and subtracting the background, the distribution of the PSNR and the RMSE remain a little extended for the images corresponding to the grains compared to those of the membrane and the carbon. Indeed, as there are many more spots in the diffraction signal of the images taken on the grains, one can attribute the remaining variations in decreasing order to the variation of intensity, radius, and position of the spots. With the registration method employed here, the intensity is slightly modified, because it is taken as the average of the spot after applying a Gaussian filter to the image. However, the errors in the position and the radius of the spots are relatively small, as shown in **Figure 4d**.

Thus, the three metrics used here designate the consecutive operations of averaging over several neighboring images and of image background subtraction as the best preparation for the registration of diffraction patterns among the options evaluated in this work.

**Macro Interface in ImageJ**


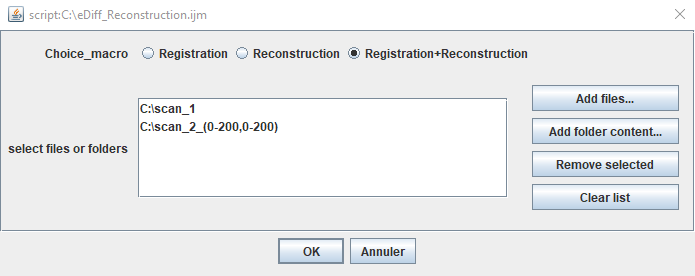


**Figure S 9** Interface of eDiff_Reconstruction ImageJ (Fiji) script: choosing a working mode and folders containing the 4D-STEM datasets as an image sequence.

**Figure S9** shows the interface window displayed when the ImageJ macro eDiff_Reconstruction.ijm is run. It allows choosing several dataset folders containing the 4D-STEM data as image sequences. Images should be sorted first by the line of scan from top to down and then by the column of scan from left to right (names like lineyyyy-columnxxxx.bmp work well). The user can choose between registration and/or registration. It is recommended for ACOM use to use both options together. The reconstruction alone can serve for example to generate one registered scan in several reconstructions with different parameters. The registration alone will be used for other in-development diffraction pattern analysis on the compressed data.

**
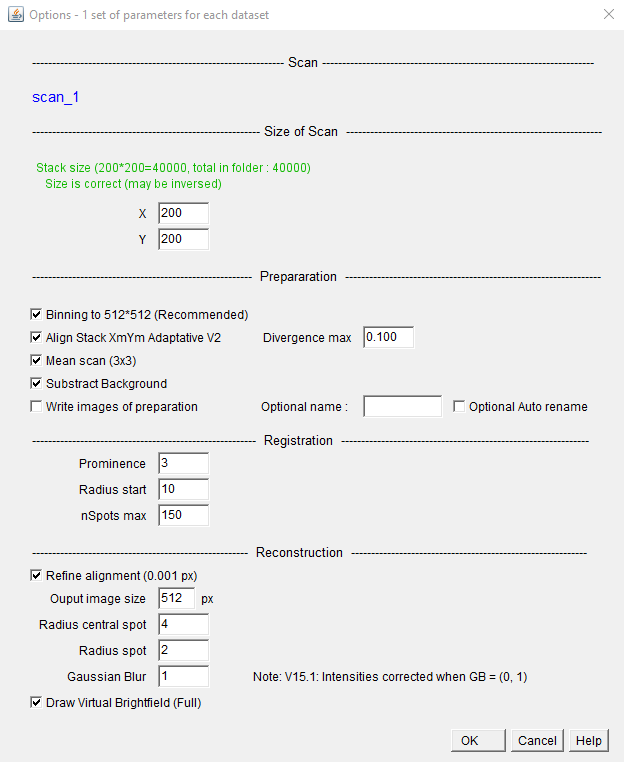
Figure S10** shows the input parameters for the registration and reconstruction proposed for each scan.

**Figure S 10** Interface displayed for each scan totreat with the option Registration+Reconstruction of eDiff_Reconstruction macro.

- Scan: Displays the title of the datasets extracted from the folder name.
- Size of Scan: 1) read the size of the scan from the folder name and compare it to 2) the total number of images in the folder, a solution of size will be proposed if 1) doesn’t correspond to 2).
- Preparation: Here are all the options to filter images before registration including a binning function to reduce to 512 px*512 px the size of images. This accelerates the process and the macro has been developed around this for format, so it’s recommended to use it (reconstruction allows to write any final size of image). Align Stack aligns diffraction pattern images by translating the grey values (with interpolation on for subpixel accuracy) to place the center of mass of the direct spot on the center of the image. Mean scan(3x3) calculates the mean over 9 neighbor images in the dataset (limit conditions: 6 images on the edge of scan, 4 images in the corner of scan). The size of the scan is conserved. Subtract background removes the mean over 50 px around each pixel of the image, it is very practical to separate the contribution of diffraction peaks on the intensity. There is an option to write the filtered images. If active, the user can choose the option to rename the image files with a custom name or with the only coordinates in scan with the auto rename option.
- Registration: Set the minimum prominence of a peak to be registered. Radius start must be smaller than the minimum distance between to spot but ideally slightly larger than the detected spot to register. It is used to refine the position, radius, and intensity of reflections registered starting from the pixel where the peak is detected. nSpots max is the maximum number of spots to register by diffraction pattern, it is used to limit the total number of reflections registered, which may be important especially if the prominence parameter is low. The extra spots ignored are the less prominent.
- Reconstruction: Refine alignment takes the registered position of the central spot (which is usually the most prominent so on top of the reflection list for each scan position), and translates all the reflections' positions to put the central spot at the exact center of image. The user can choose to force the radius value of central spots and all reflections to a constant radius. This option exists because of a lack of accuracy in the radius determination of the reflections, this accuracy on radius decreases when the intensity of reflections registered is weaker. The Gaussian blur gives back a Gaussian shape to the reflection (filter over 0 or 1 px recommended). The intensity of the original images will be retrieved on the reconstructed image with this option, using a calibration of intensity for each parameter used to correct the registered intensity. A virtual brightfield can be drawn with no additional cost by summing all the reflection intensities for each scan position.


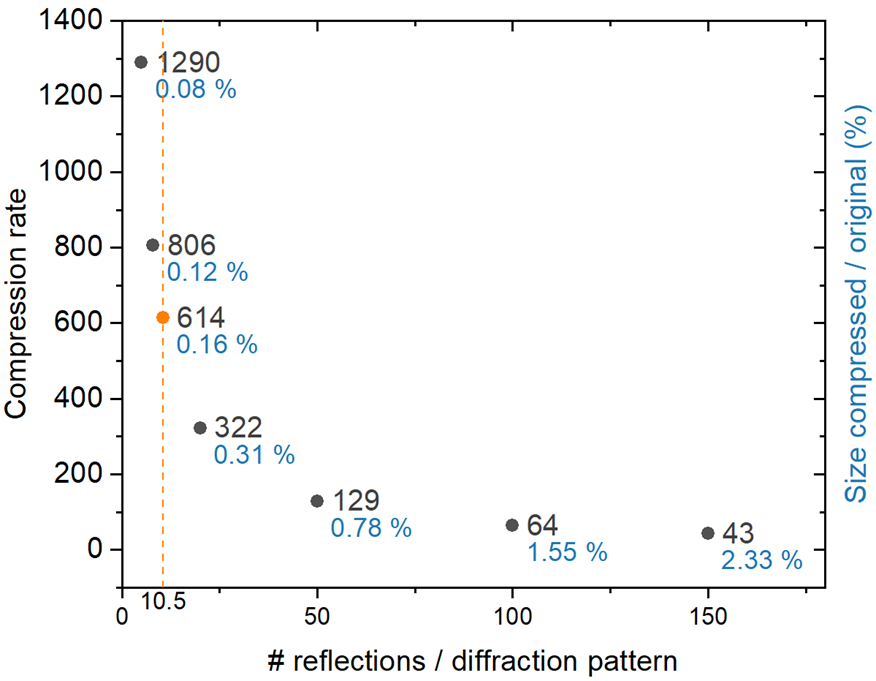


**Figure S 11** The data reduction rate and the size ratio before/after compression according to the number of reflections per diffraction pattern. The dataset reconstructed in this work contains on average 10.5 reflections per pattern and has a compressed rate of 614.


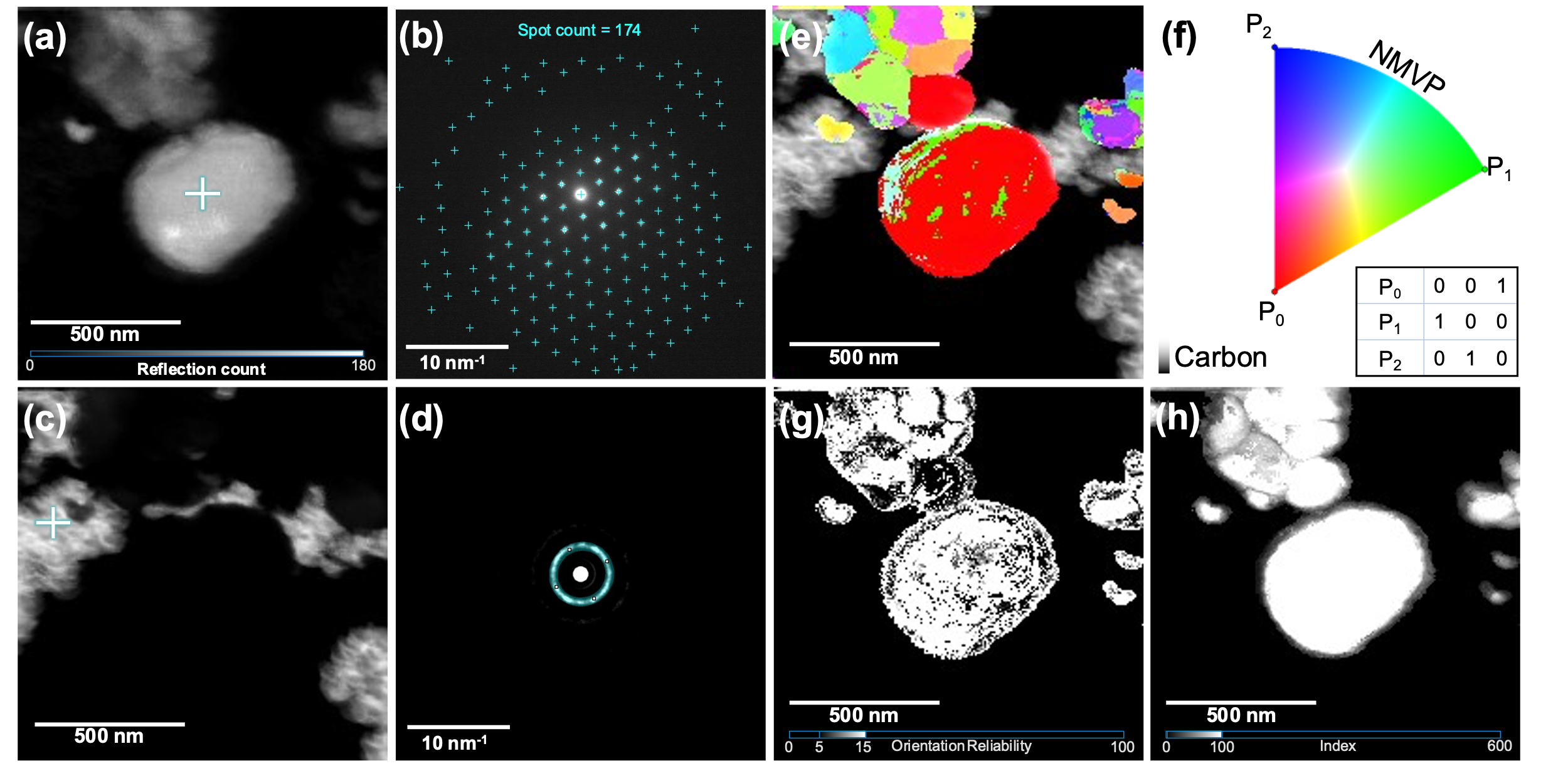


**Figure S 12** Methods to mask the artifacts due to filtering. (b) Count of diffraction spots in a DP and (a) corresponding map on a scan. (d) Integration of amorphous disk to build (c) the virtual dark field (VDF) of carbon. (e) Superposition of Astar grain orientation map and dark field amorphous map. (f) Color code for crystalline orientations. (g,h) Corresponding thresholded orientation reliability and index maps respectively. The same orientation reliability threshold has been applied on the orientation map (e).


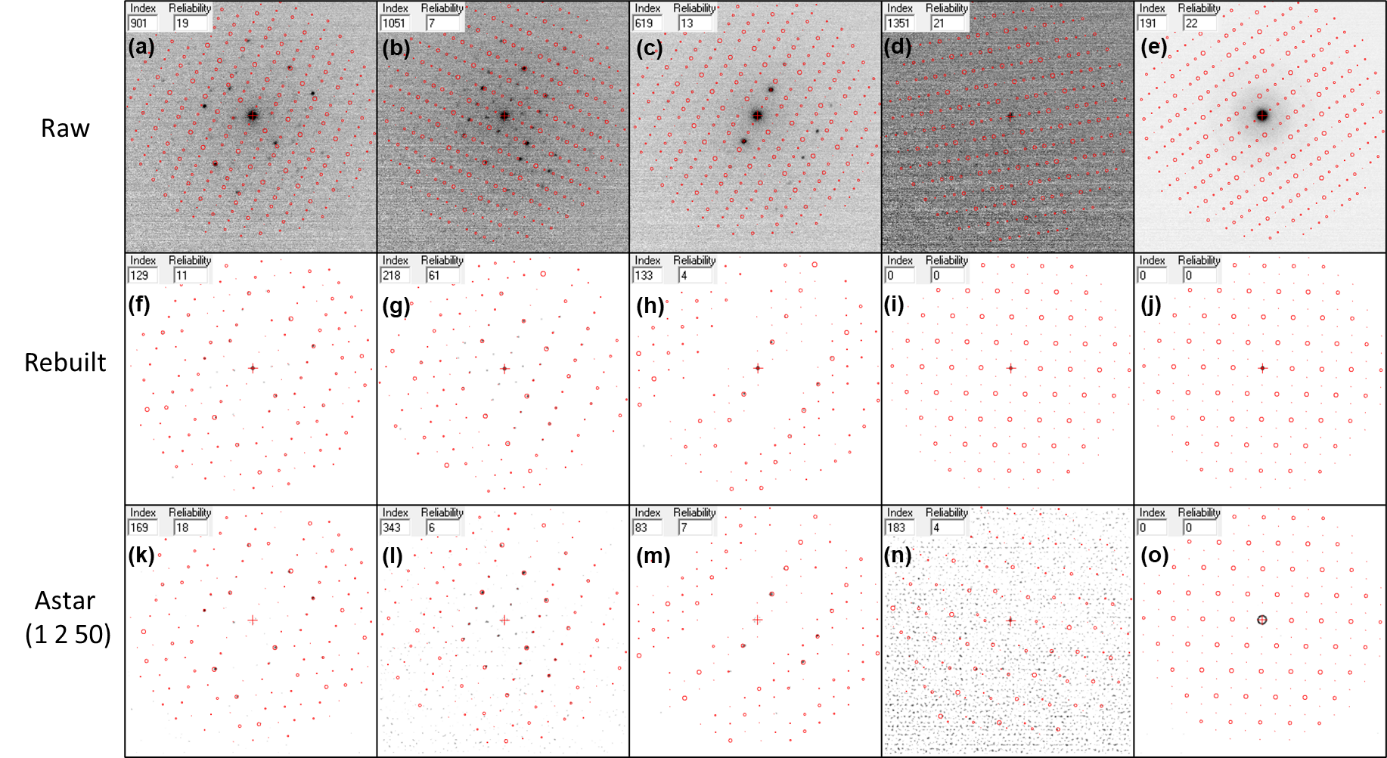


**Figure S 13** Comparison of some (a-e) Raw, (f-j) Rebuilt, and (k-o) Astar (1 2 50) diffraction patterns from the PbI_2_ dataset with the template matching solution. The Astar parameters are optimized manually to maximize the reliability of most patterns possible.

As shown in Figure S 13, the reliability and index for all raw DPs (a-e) show as expected large overfitting with too high index and reliability values regarding the poor overlapping of the templates with the DPs. The rebuilt DPs result in various reliability values that fall to 0 when no spot is present in the raw DP, as does the index. As for the rebuilt dataset, the Astar-filtered DPs present coherent fits with the templates, except for (n). The (n) matching shows a high index and reliability > 0 while the raw and rebuilt DP shows no spots, meaning that this DP is overfitting with these Astar filter parameters. For obvious statistical reasons, these few DPs can’t be quantitatively representative of the Index and Reliability values found in the dataset. However, the adaptive aspect of the registration gives the rebuilt pattern the ability to limit overfitting, which the Astar filter parameters can’t achieve as well in regions with too variable noise, as the set of parameters is fixed for the whole scan.


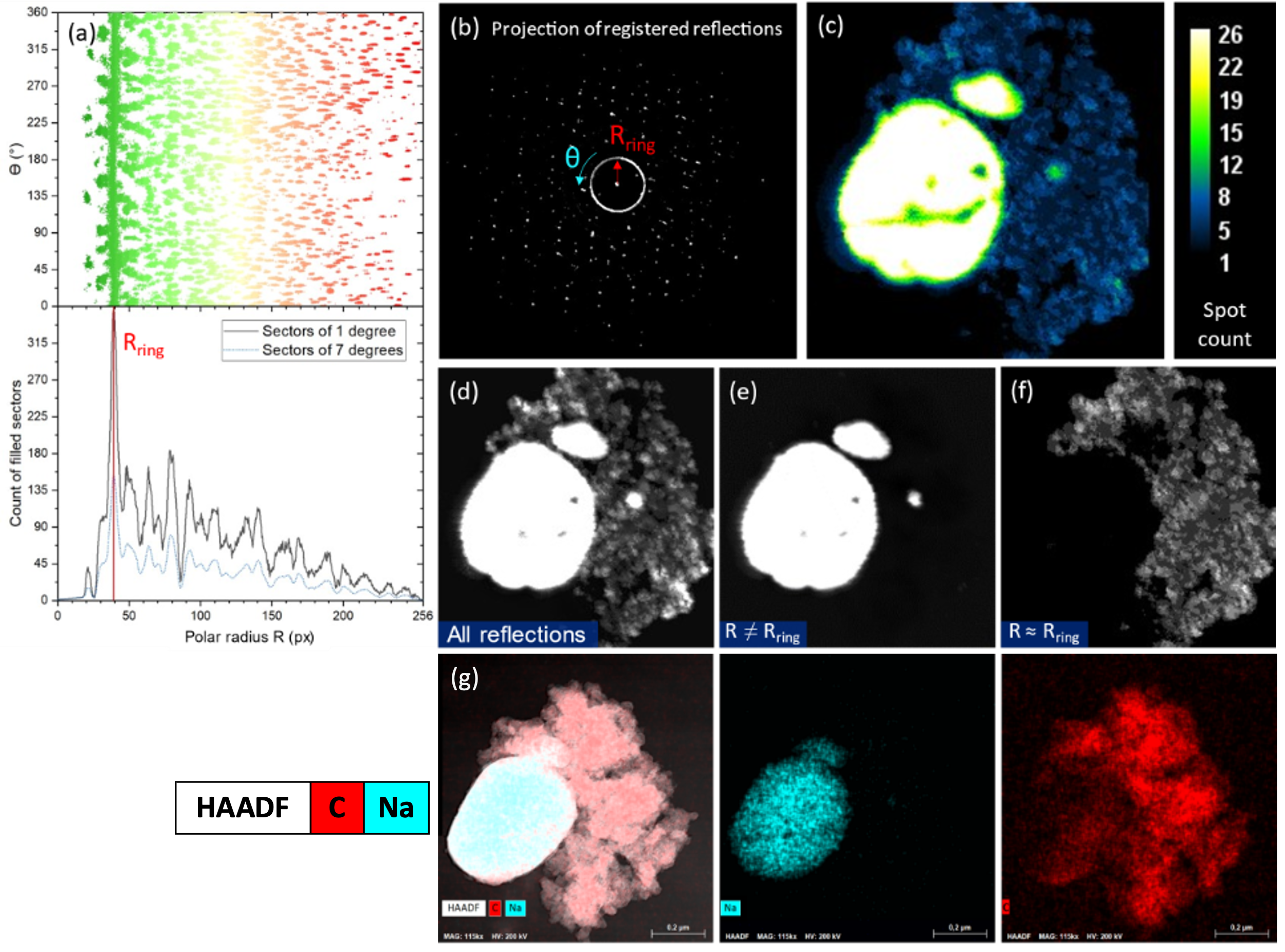


***Figure S 14*** *Mapping of the amorphous phase using a metric of annular completion. (a, top) Projection of all the registered reflections of a scan in the polar coordinates (R, θ) and (a, bottom) count of filled angle sectors indicating the angular completion according to the radius R. (b) Projection of all the registered reflections of the same scan in cartesian coordinates (x, y). (c) Spot count map of the same scan using all registered reflections. Projection of reflections intensity (virtual dark field) of (d) All registered reflections, (e) reflections which radius is different from the detected ring R, (f) reflections belonging to the radius ring R. (g) HAADF-EDX maps of Na and carbon on tilted sample.*

As shown in **Figure S14**, the automatic detection of the amorphous phase ring uses a metric of angular completion. The count of angular sectors (1 degree) containing at least one reflection indicates how complete is a ring. By default, the radius R of the amorphous ring is found at the maximum of angular completion, as shown in **Figure S14a**. As shown in **Figure S14b**, the projection of all reflections reveal a clearly defined ring, that can be eventually manually spotted to separate the amorphous and crystalline data. However, the automation of ring detection allows an accurate detection with less points contained in the ring, and lowers the user bias in a manual definition of the ring radius. The spot count map shown in **Figure S14c** reveals that a threshold in spot count is not accurate enough to separate crystalline and carbon regions, especially when there is superposition of the two phases, as displayed in HAADF-EDX maps in **Figure S14g**. Note that the HAADF-EDX maps are tilted of ~20 degrees compared to 4D-STEM maps to optimize the EDX signal. On the other hand, the issue of superprosition of amorphous and crystalline phases is reduced after using the ring detection, as shown in the virtual dark fields of mixed and separated amorphous and crystalline data of **Figure S14d, e, and f**. Indeed, the only remaining issue after the separation of crystalline and amorphous phases is the relatively rare case when some reflections belonging to the diffraction pattern of a crystal are assimilated to the amorphous phase, because their position is at the same radius than the amorphous ring. However, this small data loss is quantitatively insignificant over a full scan considering the number of reflection registered. Besides, this qualitatively only implies a loss of true positive in the registered crystalline data, thus without affecting the template matching with false positives, which is coherent with the main intention in this work.

Overall, this technique has proven to be efficient for our data, but it is important to note that the technique efficiency would decrease with high polycrystallinity, which would imply the presence of other rings in the projection, requiring either the use of manual determination or more elaborated algorithms.

Some ImageJ’s plugins have been used and implemented In this work :

- Joachim Walter's FFT Filter plugin:

<http://rsb.info.nih.gov/ij/plugins/fft-filter.html>

- SNR, PSNR, RMSE, MAE plugin to assess the quality of images written by Daniel Sage at the Biomedical Image Group, EPFL, Switzerland:

<http://bigwww.epfl.ch/sage/soft/snr/>

- Find Maxima contributed by Michael Schmid:

<https://imagej.nih.gov/ij/docs/menus/process.html>

<https://imagej.nih.gov/ij/developer/api/ij/ij/plugin/filter/MaximumFinder.html>

- Rolling Ball Subtract background by Stanley Sternberg

"Biomedical Image Processing", IEEE Computer, January 1983.

DOI: 10.1109/MC.1983.1654163

[https://imagej.net/ij/docs/menus/process.html#background](https://imagej.net/ij/docs/menus/process.html%23background)
